# Elevated Fibronectin Levels in Profibrotic CD14^+^ Monocytes and CD14^+^ Macrophages in Systemic Sclerosis

**DOI:** 10.3389/fimmu.2021.642891

**Published:** 2021-08-24

**Authors:** Michał Rudnik, Amela Hukara, Ievgeniia Kocherova, Suzana Jordan, Janine Schniering, Vincent Milleret, Martin Ehrbar, Karin Klingel, Carol Feghali-Bostwick, Oliver Distler, Przemysław Błyszczuk, Gabriela Kania

**Affiliations:** ^1^Department of Rheumatology, Center of Experimental Rheumatology, University Hospital Zurich, University of Zurich, Zurich, Switzerland; ^2^Department of Obstetrics, University Hospital Zurich, Zurich, Switzerland; ^3^Department of Molecular Pathology, University Hospital Tuebingen, Tuebingen, Germany; ^4^Division of Rheumatology, Medical University of South Carolina, Charleston, SC, United States; ^5^Department of Clinical Immunology, Jagiellonian University Medical College, Krakow, Poland

**Keywords:** systemic sclerosis, CD14+ monocytes, CD14+ macrophages, fibrosis, fibronectin, TGF-β

## Abstract

**Background:**

Systemic sclerosis (SSc) is an autoimmune disease characterized by overproduction of extracellular matrix (ECM) and multiorgan fibrosis. Animal studies pointed to bone marrow-derived cells as a potential source of pathological ECM-producing cells in immunofibrotic disorders. So far, involvement of monocytes and macrophages in the fibrogenesis of SSc remains poorly understood.

**Methods and Results:**

Immunohistochemistry analysis showed accumulation of CD14^+^ monocytes in the collagen-rich areas, as well as increased amount of alpha smooth muscle actin (αSMA)-positive fibroblasts, CD68^+^ and mannose-R^+^ macrophages in the heart and lungs of SSc patients. The full genome transcriptomics analyses of CD14^+^ blood monocytes revealed dysregulation in cytoskeleton rearrangement, ECM remodeling, including elevated *FN1* (gene encoding fibronectin) expression and TGF-β signalling pathway in SSc patients. In addition, single cell RNA sequencing analysis of tissue-resident CD14^+^ pulmonary macrophages demonstrated activated profibrotic signature with the elevated *FN1* expression in SSc patients with interstitial lung disease. Peripheral blood CD14^+^ monocytes obtained from either healthy subjects or SSc patients exposed to profibrotic treatment with profibrotic cytokines TGF-β, IL-4, IL-10, and IL-13 increased production of type I collagen, fibronectin, and αSMA. In addition, CD14^+^ monocytes co-cultured with dermal fibroblasts obtained from SSc patients or healthy individuals acquired a spindle shape and further enhanced production of profibrotic markers. Pharmacological blockade of the TGF-β signalling pathway with SD208 (TGF-β receptor type I inhibitor), SIS3 (Smad3 inhibitor) or (5Z)-7-oxozeaenol (TGF-β-activated kinase 1 inhibitor) ameliorated fibronectin levels and type I collagen secretion.

**Conclusions:**

Our findings identified activated profibrotic signature with elevated production of profibrotic fibronectin in CD14^+^ monocytes and CD14^+^ pulmonary macrophages in SSc and highlighted the capability of CD14^+^ monocytes to acquire a profibrotic phenotype. Taking together, tissue-infiltrating CD14^+^ monocytes/macrophages can be considered as ECM producers in SSc pathogenesis.

## Introduction

Systemic sclerosis (SSc) is an autoimmune disease, characterized by high morbidity and mortality and a significant reduction in quality of life. Microvascular damages, dysregulation of innate and adaptive immunity and multiorgan fibrosis are implicated in the pathophysiology of SSc (1). SSc is characterized by a patient-to-patient variability in clinical manifestations, autoantibody profiles, extent of disease, treatment response and reduced survival rate (2). Changes in internal organ architecture, leading to pulmonary and cardiac complications and dysfunction remains the major causes of deaths among SSc patients (3).

Fibrogenesis is a multistage process, considered as the result of impaired tissue repair responses, in which abnormal production of cytokines, growth factors and angiogenic factors turn tissue homeostasis towards the excessive accumulation of extracellular matrix (ECM) (4). Fibrosis is usually an outcome of prolonged and exaggerated activation of fibroblasts, which differentiate into myofibroblasts (5). In contrast to physiological wound healing, in which myofibroblast are present only transiently, in fibrosis myofibroblasts become a permanent cellular component of the tissue (6).

Myofibroblasts are characterized by expression of alpha smooth muscle actin (αSMA) forming stress fibers and exhibit an increased capacity to synthesize collagens, fibronectin, and other ECM components. Fibronectin is a high-molecular weight glycoprotein of the extracellular matrix, which binds to integrins and other extracellular matrix proteins such as collagen, fibrin, and heparan sulfate proteoglycans (7). Increased deposition of fibronectin, paralleled to accumulated collagen, has been reported in the SSc skin (8, 9).

The cellular source of myofibroblasts in wound healing and fibrotic lesions is still debatable. Although most evidence point to resident fibroblasts, other tissue-resident cell types or cells recruited from circulation have been shown to acquire a myofibroblast-like phenotype (10). In experimental models of lung and kidney fibrosis, bone marrow-derived fibrocytes have been shown to be recruited into injured or fibrotic tissues in response to chemokine signals, where they differentiate into fibroblasts or myofibroblasts (11). Lineage tracing experiments have provided convincing evidence that pericytes are an important source of myofibroblasts during renal fibrogenesis in animal models, and these cells might also be a source of myofibroblasts in SSc (12, 13). During fibrogenesis, epithelial cells have been hypothesized to undergo epithelial-to-mesenchymal transition and transdifferentiate into myofibroblasts in response to TGFβ and other profibrotic cytokines. Additionally, SSc tissues are chronically hypoxic, which can further promote the formation of myofibroblast-like cells (14). In addition to epithelial cells, endothelial cells have also been hypothesized to transdifferentiate into myofibroblasts through endothelial-mesenchymal transition (15).

The primary mechanism underlying excessive fibrosis in SSc remains unknown; however, TGFβ is one of the best-studied mediators regulating fibrotic processes, including fibroblast differentiation, ECM deposition and tissue contraction (16). Elevated levels of TGFβ-regulated genes (cartilage oligomeric matrix protein, thrombospondin-1) were reported in the skin lesions from SSc patients (17). TGFβ signalling regulates the expression of profibrotic genes mostly *via* the SMAD-dependent canonical pathway (18). However, the involvement of several non-canonical pathways in fibrotic processes was acknowledged. For example, activation of the ERK-MAPK signalling pathway leads to upregulation of type I collagen in SSc fibroblasts (19). Ultimately, inhibition of TGFβ signalling has been addressed as a potential treatment strategy; however, conflicting results were reported following TGFβ blockade (20).

Myeloid cells, including monocytes, have been shown to be essential regulators of fibrosis as producers of chemokines, inflammatory cytokines and growth factors (21, 22). Our group has previously reported that the microenvironment of inflamed lung induces myeloid progenitors to acquire a myofibroblast phenotype (23). Similarly, we showed that heart-infiltrating myeloid cells served as myofibroblast progenitors in the model of post-inflammatory cardiac fibrosis (24). In the present study, we evaluated the differentiation potential of circulating CD14^+^ monocytes from healthy controls and SSc patients into a myofibroblast-like phenotype.

## Material and Methods

### SSc Patients and Healthy Controls

Human blood samples and skin biopsies collection were approved by the local ethics committee of the Canton Zurich (KEK-ZH 515, PB-2016-02014, KEK-Nr 2018-01873). All study subjects provided written informed consent. SSc patients and healthy controls were recruited at the Department of Rheumatology of University Hospital Zurich. All the patients fulfilled the ACR/EULAR 2013 classification criteria for SSc.

The patients diagnosed with SSc by experts with Raynaud syndrome and a least one other SSc characteristic such as: SSc specific antibodies, SSc characteristic capillaroscopy changes and/or puffy fingers. The patients not fulfilling the ACR/EULAR 2013 classification criteria for SSc were grouped as early-SSc. The patients fulfilling the criteria were further divided into lcSSc and dcSSc subgroups, according to Le Roy et al. (25). Detailed demographics and clinical characteristics of SSc patients are included in **Table 1**. Healthy control group was age- and gender matched (age mean=44.4 ± 10.8, female 17/20 (85%).

**Table 1:** Definitions of items and organ manifestation are according to EUSTAR.

Baseline demographic and clinical characteristics of the SSc patients	
	SSc pat (N = 37)
**Demographics**	
Age (mean ± SD)	54.0 ± 14.6
Female sex	32/37 (86.5%)
Disease duration (mean ± SD; years)	14.7 ± 12.5
ACR/EULAR criteria fulfilled	28/37 (75.7%)
Subtype	
Diffuse SSc	4/37 (10.8%)
**Skin/Vascular**	
Raynaud’s Phenomenon	32/37 (86.5%)
Digital ulcers	12/37 (32.4%)
Active digital ulcers	3/35 (8.6%)
Pitting scars	11/35 (31.4%)
Scleredema	26/35 (74.2%)
Telangiectasia	20/37 (54.1)
mRSS (mean ± SD)	2.8 ± 4.1
Abnormal nailfoldcapillaroscopy	30/33 (90.6%)
**Musculoskeletal**	
Tendon friction rubs	1/35 (2.9%)
Joint synovitis	5/36 (13.9%)
Joint contractures	9/32 (42.0%)
Muscle weakness	0/31 (0)
**Gastrointestinal**	
Esophageal symptoms	20/37 (54.1%)
Stomach symptoms	8/33 (24.2%)
Intestinal symptoms	14/33 (42.4%)
**Cardiopulmonary**	
Dyspnea	
Stage 1	27/34 (79.4%)
Stage 2	5/34 (14.7%)
Stage 3/4	2/34 (5.9%)
Diastolic dysfunction	8/31 (25.8%9
Pericardial effusion	3/24 (12.5%)
Conduction blocks	2/35 (5.7%)
LVEF<45%	0/35 (0)
PAH by RHC	0/37 (0)
Lung fibrosis on HRCT	11/37 (29.7%)
FVC, % predicted (mean ± SD)	93.5 ± 15.4
FVC<70% predicted	1/37 (2.7%)
FEV, % predicted (mean ± SD)	93.2 ± 14.6
TLC, % predicted (mean ± SD)	103.5 ± 16.6
DLCO, % predicted (mean ± SD)	76.0 ± 18.8
DLCO<70% predicted	10/33 (30.3%)
**Kidney**	
Renal crisis	1/37 (2.7%)
**Laboratory parameters**	
ANA positive	37/37 (100%)
ACA	22/37 (59.5%)
Anti-Scl-70 positive	8/36 (22.2%)
Anti-U1RNP positive	0/37 (0)
Anti RNA-polymerase III positive	2/36 (5.5%)
Creatinine kinase elevation	2/27 (7.4%)
Proteinuria	2/35 (5.7%)
Hypocomplementaemia	2/36 (5.5%)
ESR>25 mm/h	9/31 (29.0%)
CRP elevation	5/26 (19.2%)
Active disease (VAI>3)	1/32 (3.1%)
Immunosuppressive therapy	5/37 (13.5%)

Data are presented as number (n)/total valid cases (N) (%). Disease duration was calculated as the difference between the date of the baseline visit and the date of the first non-Raynaud’s symptom of the disease as reported by the patient. Pulmonary hypertension was judged on RHC. Active disease was defined as a score >3 by calculating European Scleroderma Study Group disease activity indices for systemic sclerosis proposed by Valentini. Immunosuppressive therapy was defined as treatment with corticosteroids (prednisone dose ≥10 mg/day or other dosage forms in equal dose) or any immunosuppressant.

ACA, anti-centromere antibody; ANA, antinuclear antibody; Anti-Scl-70, anti-topoisomerase antibody; CRP, C reactive protein; HRCT, computed tomography; DLCO, diffusing capacity for carbon monoxide; ESR, erythrocyte sedimentation rate; FEV1, forced expiratory volume in 1 sec; FVC, forced vital capacity; LVEF, left ventricular ejection fraction; mRSS, modified Rodnan skin score; NYHA, New York Heart Association; TLC, total lung capacity; VAI, Valentini activity index.

Human endomyocardial biopsies were provided by the University Hospital Tubingen, Germany. Samples were obtained from SSc patients with cardiac involvement (inflammatory dilated cardiomyopathy) and controls (patients with healed myocarditis). Lung biopsies were provided by the Division of Rheumatology, Medical University of South Carolina, Charleston, USA. The samples were obtained from SSc-related interstitial lung disease (SSc-ILD). Sample tissue from downsized lung transplants from healthy individuals served as controls. The experiments with re-use of human material were approved by Swissethics (KEK-Nr 2019-00058, KEK-Nr 2018-01873) and were performed in conformity with the principles outlined in the Declaration of Helsinki. Transcriptomic analysis of an already published dataset of human lung tissues has been included. For this, the University of Pittsburgh Institutional Review Board approved ethics of use the human lung samples as previously described (26).

### CD14^+^ Monocytes Isolation and Differentiation

Blood samples were collected in EDTA tubes (BD Vacutainer) and processed within 24h. Peripheral blood mononuclear cells (PBMCs) were isolated by gradient centrifugation on cell separation medium (Lympholyte, Cedarlane), followed by magnetic-activated cell sorting for CD14 using human microbeads and AutoMACS Pro device (Miltenyi Biotec). Cells were cultured in DMEM low glucose medium (Sigma) with the addition of 10% Foetal Bovine Serum (FBS) and 1% penicillin/streptomycin (both Gibco). To differentiate into myofibroblast-like cells, CD14^+^ monocytes were stimulated with 10 ng/ml TGFβ (PeproTech), 10 ng/ml IL-4, 10 ng/ml IL-10 and 10 ng/ml IL-13 (Immunotools) for 7 days.

### RNA Extraction

Total RNA was isolated using the Quick-RNA Microprep isolation kit (Zymo Research). Directly after monocyte treatment, cells were washed with PBS and lysed in RNA lysis buffer (Zymo Research). An equal volume of absolute ethanol (Millipore) was added and mixed. Lysates were further processed on the columns. Genomic DNA was removed by DNase I treatment. RNA was washed twice and eluted in 10-15 μl of nuclease-free water (Promega). RNA concentration and purity were assessed on NanoDrop 1000 (Thermo Fisher Scientific).

### Bulk and Single Cell (sc) RNA Sequencing and Data Analysis

For RNA sequencing, RNA was isolated from SSc patients and healthy controls [as described previously in **Supplementary Table 1** in (27)] as described above, and RNA Integrity Number (RIN) was assessed by Tape Station (Agilent). Samples with RIN≥8 were further processed. RNA sequencing was performed by the Functional Genomics Centre Zurich. From 100 ng of total RNA, polyA libraries were prepared using the Illumina TruSeq RNA Stranded mRNA library Kit. Sequencing was performed on the Illumina HiSeq 4000 platform. Quality of the sequencing was controlled by FastQC package. Reads were aligned to the genome using the STAR algorithm. Gene expression profiles were next calculated by the FeatureCount algorithm. For differentially expressed genes, the DeSEQ2 algorithm was used with a threshold of minimum 10 reads for a transcript to be considered as present. Pathway enrichment analysis of differentially expressed genes (p ≤ 0.01, |log_2_ratio| ≥0.5) was performed by the Metacore software, as described previously (27).

Human explanted lung tissues were digested and scRNA-sequencing was performed as previously described (26).

### ScRNAseq Analysis of Each Sample

Raw count matrices produced from CellRanger were loaded from the dataset GSE128169. Empty droplets were distinguished from droplet-containing cells by using the emptyDrops function from the R package DropletUtils (28, 29). Only droplets that obtained an FDR <= 0.001 were called as cells. Doublets were also discarded from further analysis using the R package scDblFinder. Low quality cells were identified based on the number of reads, genes and mitochondrial content using the R package scuttle. We excluded cells that were outliers according to at least one of the following thresholds: number of reads < 3 MADs, number of gene < 3 MADs, mitochondrial content > 3 MADs. Additionally, we discarded genes with < 1 UMI count in < 0.01 of the remaining cells. We used the SCTransform (30) method from the R package Seurat (31) to normalize and scale the data.

### Integration of Multiple Samples

Multiple samples were combined using the Seurat integration workflow (32). After integration we reduced the dimensionality of the data using Principal Component Analysis (PCA). The main 30 principal components were used to perfor unsupervised clustering with a resolution value of 0.8. After clustering and visualization with Uniform Manifold Approximation and Projection (UMAP), cell populations were identified through examination of gene markers in the associated transcriptome.

Cells that had > 0 counts for the CD14 gene were called as CD14^+^ cells. All plots and downstream analysis were performed using Seurat.

### RT-qPCR

For reverse transcription, 200-300ng of total RNA was used. The reaction was performed using MultiScribe reverse transcriptase (Thermo Fisher Scientific), random hexamers and RNAase inhibitor (both Roche). Subsequently, the qPCR reaction was performed using the SYBR green GoTaq qPCR master mix (Promega) on Agilent Stratagene Mx3005P qPCR instrument. Sequences of primers are listed in **Supplementary Table 1**. Relative gene expression was calculated using the 2^-ΔΔCt^ method. *GAPDH* was used as the reference gene.

### Protein Extraction and Immunoblotting

After the stimulation with cytokines, cells were washed once with ice-cold PBS, collected, centrifuged and lysed for 30 minutes on ice in RIPA buffer (Sigma) supplemented with proteases and phosphatases inhibitors (Roche). An equal amount of the protein was loaded and separated by SDS-PAGE electrophoresis, followed by wet transfer on the nitrocellulose membrane (GE Healthcare). The membrane was further incubated for 1 hour in blocking buffer (5% BSA in TBS-T). Further, membranes were probed overnight with primary antibodies, listed in **Supplementary Table 2**, in blocking buffer at 4°C. Further, membranes were incubated for 1 hour at room temperature with secondary HRP-conjugated antibodies. Signal was developed with ECL substrate (SuperSignal West Pico PLUS, Thermo Scientific) and acquired on the Fusion fx (Vilber) device.

### ELISA

For the detection of human pro-collagen 1α1 DuoSet ELISA (RnD Systems) was used according to the manufacturer’s protocol. Briefly, 96-well plates were coated with capture antibodies overnight in room temperature and further blocked with 2% BSA in phosphate-buffered saline (PBS) supplemented with 0.05%Tween 20. Between each step, plates were washed three times with PBS. The protein standards and samples were applied and incubated for 2 hours. Next, plates were incubated with biotin-conjugated detection antibodies for 2 hours and streptavidin-HRP for 30 minutes in room temperature. Signal was developed with TMB substrate (Thermo Scientific), and 450 nm absorbance was measured on a BioTEK HT plate reader (Tecan). Concentrations were calculated according to the respective standard curves.

### Treatment With Pharmacological Inhibitors and Cytotoxicity Assessment

SD208 [1 μM], SIS3 [2 μM], (5Z)-7-Oxozeaenol (OXO) [1 μM] pharmacological inhibitors used in the project were purchased from Tocris Biosciences. To determine optimal non-toxic concentrations, we performed toxicity tests. CD14^+^ monocytes were incubated with 2-fold dilutions of inhibitors starting from 5 or 10 µM. Cells were incubated for 24h, stained with propidium iodide (Biolegend), and cytotoxicity was evaluated by flow cytometry. The highest non-toxic concentration was used in further experiments.

### 2D and 3D Co-Culture With Dermal Fibroblasts

To distinguish cells in both systems, monocytes were stained with Cell Trace Violet (Thermo Scientific), and fibroblasts were stained with CFSE (Biolegend) according to the manufacturer’s protocol. For 2D co-cultures, fibroblasts were plated 24h prior to the addition of monocytes. Cells were cultured for 7 days and sorted using FACSAria III cell sorter.

For the 3D co-culture model, 3DProSeed^®^ hydrogel microtiter plate (Ectica Technologies) were used. Firstly, labelled fibroblasts were plated and allowed to penetrate hydrogels for 24h. Next, monocytes were added. Plates were incubated for 7 days, and anti-αSMA/phalloidin (both Sigma) staining was performed. Co-cultures were visualized using Leica SP8 confocal microscope.

### Immunohistochemistry, Imaging and Quantification

Collected tissue samples were washed in PBS and fixed for 16 hours in 4% paraformaldehyde in PBS. Next, tissues were rinsed in distilled water and transferred to 50% ethanol. Biopsies were then dehydrated (three incubations in 80% ethanol for 1 hour, three incubations in 96% ethanol for 1 hour, two incubations in 100% ethanol for 1 hour). Tissues were cleared twice in xylene for 1 hour and subsequently incubated twice in a 56°C paraffin bath for 3 hours. After paraffin embedding, 4 μm thick sections were placed on Superfrost Plus slides (Thermo Scientific) and dried overnight. Lung sections were cut at a thickness of 4 μm and stained with hematoxylin and eosin (HE) for analysis of the lung architecture and the presence of cellular infiltrates, and with Picrosirius Red to detect collagen deposition using standard protocols (33).

For immunohistochemistry sections were deparaffinized in xylene for 10 minutes (3 times) and rehydrated by sequential incubations in ethanol solutions (100%, 100%, 96% and 80%) for 3 minutes each. Sections were eventually washed for 5 minutes in distilled water. Antigen retrieval was performed in citrate buffer (10 mM citrate, 0.05% Tween, pH=6), and incubated at 95°C for 15 minutes. Endogenous peroxidases were blocked by 3% H_2_O_2_ solution for 15 minutes. Unspecific antibody binding was blocked with 10% goat serum in Background Reducing Antibody Diluent (Dako). Endogenous biotin was blocked by Avidin-Biotin Block kit (Vector Laboratories). Sections were incubated with primary antibodies, listed in the **Supplementary Table 2**, in 4°C overnight. The appropriate biotinylated secondary antibodies (Vector Laboratories) were incubated for 30 minutes at room temperature, followed by 30 minutes incubation with VECTASTAIN Elite ABC kit (Vector Laboratories). Staining was developed using Vector DAB or Vector Red (Vector Laboratories) followed by a counterstaining of nuclei for 1 minute in Mayer’s hematoxylin solution (J.T Baker). All sections were mounted using Pertex mounting medium (Dako). CD14-collagen-1 was performed by Sophistolab AG.

Full slide images were acquired using a Zeiss Axio Scan Z1 slide scanner. ImageJ software was used for relative quantification of the signal in the sections. For CD45, mannose-R, CD86, iNOS and arginase-1 staining analysis, the “HDAB” plug-in was applied, while for Picrosirius Red and α-SMA “Fast Red, Fast Blue” plug-in was used. Deconvoluted images were used to calculate the area of the nuclear staining and the area of the specific signal. The value for each section was calculated as the ratio of the specific signal to nuclei signal.

### Statistical Analysis

Statistical analysis was performed using GraphPad Prism 8 software. Data distribution was calculated using the Shapiro-Wilk test. Normally distributed data were presented as mean ± standard deviation and analyzed by unpaired two-tailed parametric *t*-test. For non-normally distributed data, unpaired non-parametric Mann-Whitney *U* test was used, and medians were presented. For comparisons of more than two groups, two-way ANOVA test with multiple comparisons (normally distributed data) and Kruskal-Wallis test with multiple comparisons (non-normally distributed data) were used. Differences were considered statistically significant for *p*<0.05. n refers to the number of biological replicates.

Additional methods are described in the **Supplementary Material**.

## Results

### Infiltration of CD14^+^ Monocytes Into the Organs Co-Localizes With Fibrosis in SSc

**Figure 1:**
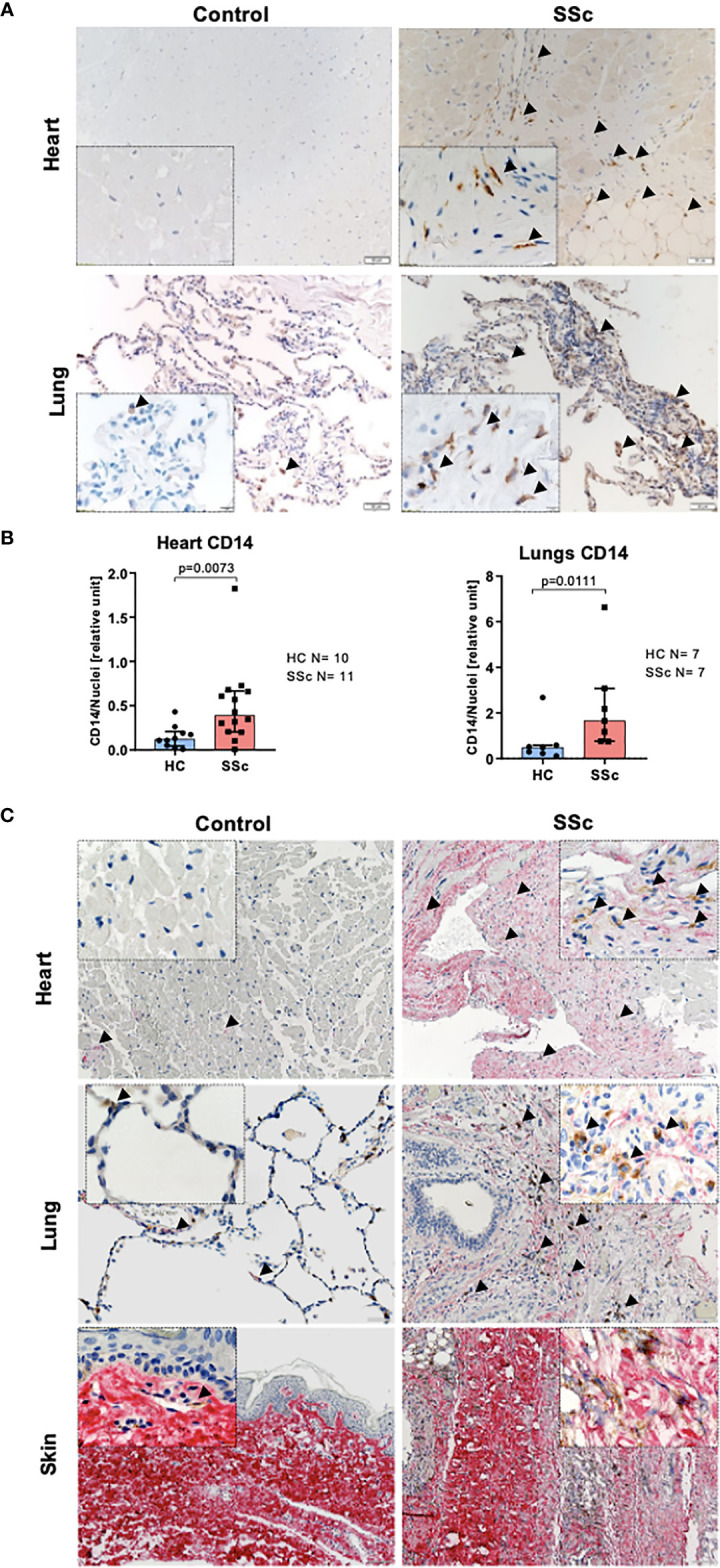
Monocyte infiltration into the fibrotic lesion in SSc tissues. **(A)** Representative images of paraffin-embedded sections of the heart and lungs, and **(B)** corresponding quantification of IHC staining for CD14 (lungs HC N=7, SSc N=7, heart HC N=10, SSc N=11, *unpaired t-test).* **(C)** Representative images of paraffin-embedded sections of the heart, lungs and skin co-stained for pro-collagen I (red) and CD14 (brown). **(A, C)** Scale bar 100μm, scale bar in insert 20μm.

**Figure 2:**
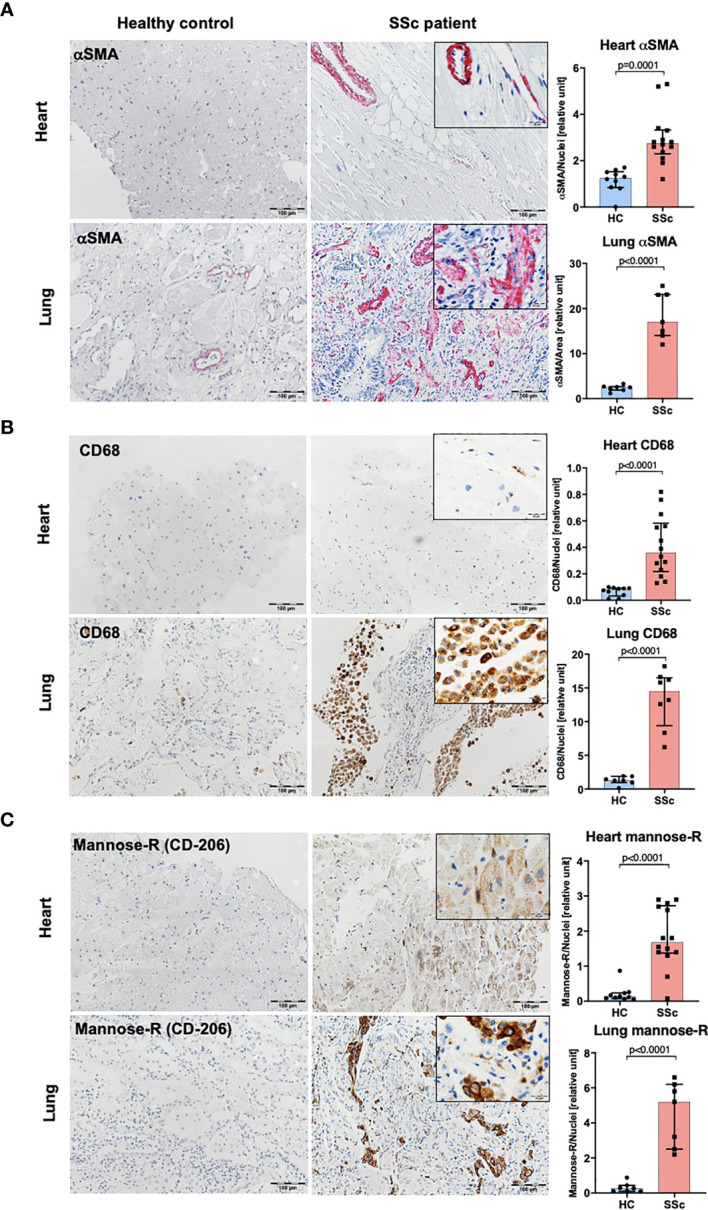
Characteristic of human SSc lung and heart tissues. Representative images of paraffin-embedded sections of the heart and lungs and corresponding quantification of IHC staining for αSMA **(A)**, CD68 **(B)** and mannose-R (CD206) **(C)** (lung HC N=7, lung SSc N=7, heart HC N=10, heart SSc N=11, *unpaired t-test*; scale bar 100μm, scale bar in insert 20μm).

In line with data published previously (34), we observed increased infiltration of CD14^+^ cells into the heart and lungs of SSc patients compared to control tissues (**Figures 1A, B**). Moreover, CD14 signal co-localized with collagen-rich lesions in different SSc tissues (**Figure 1C**). This data suggests that monocytes may participate in fibrotic processes in many organs during pathological tissue remodeling in SSc. Furthermore, heart and lungs of SSc patients showed the increased levels of αSMA^+^ (myo)fibroblasts, CD68^+^ pro-inflammatory macrophages and mannose-R^+^ (CD206) alternatively activated macrophages (**Figures 2A–C**) that reflects inflammatory and fibrotic microenvironment in different SSc tissues.

### Activation of Profibrotic Pathways in CD14^+^ Blood Monocytes in SSc

**Figure 3:**
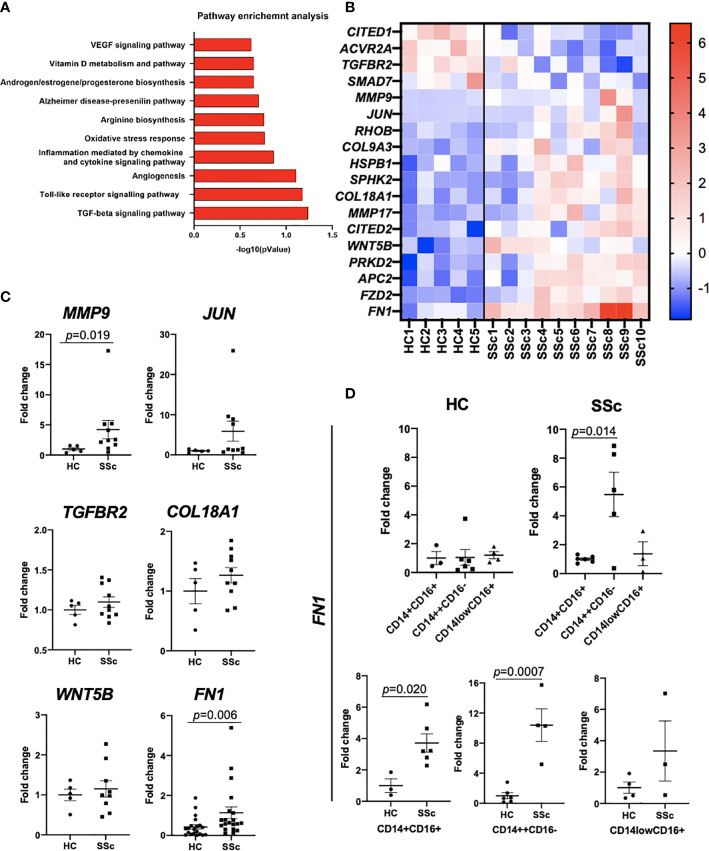
Transcriptomic analysis of CD14^+^ monocytes from SSc patients and HC. **(A)** Pathway enrichment analysis of SSc-related biological processes calculated based on differentially expressed gene sets (Metacore software). **(B)** Differentially expressed genes involved in fibrosis from RNAseq data. **(C)** qPCR confirmatory analyses of selected genes from RNAseq data (N=5-20, *Mann Whitney-U-test)*. **(D)** qPCR analysis of *FN1* mRNA levels in sorted CD14^++^CD16^-^, CD14^low^CD16^+^ and CD14^+^CD16^+^ monocyte from SSc patients and HC (n=3-6, *Mann Whitney-U-test*).

We compared the transcriptome profiles of the total pool of SSc CD14^+^ monocytes in relation to CD14^+^ monocytes obtained from healthy controls (HC). Pathway analysis revealed enrichment of SSc and fibrotic-associated processes, such as “TGF-beta signalling pathway”, “Toll-like Receptor Signalling Pathway”, “Angiogenesis” and “VEGF signalling pathway” in SSc CD14^+^ monocytes (**Figure 3A**). At the transcription level, we observed dysregulated expression of essential profibrotic genes: *TGFBR2, MMP9, JUN, COL9A3, COL18A1, FN1* and *WNT5B* (**Figures 3B, C**). Further, we demonstrated that SSc CD14^++^CD16^-^ monocyte population showed significantly higher *FN1* expression than SSc CD14^+^CD16^+^ and CD14l^ow^CD16^+^ monocyte populations (**Figure 3D**). Of note, we did not notice any difference in *FN1* expression between these three monocyte populations in HC monocytes (**Figure 3D**). Importantly, SSc CD14^++^CD16^-^ and CD14^+^CD16^+^ monocyte populations revealed profibrotic features by expressing upregulated *FN1* levels compared to HC monocytes (**Figure 3D**).

This result indicated an activated, profibrotic phenotype of SSc CD14^+^ monocytes in the circulation, which might predispose them to acquire pathogenic phenotype in the fibrotic tissue.

### Activation of Profibrotic Pathways in CD14^+^ Pulmonary Macrophages in SSc

**Figure 4:**
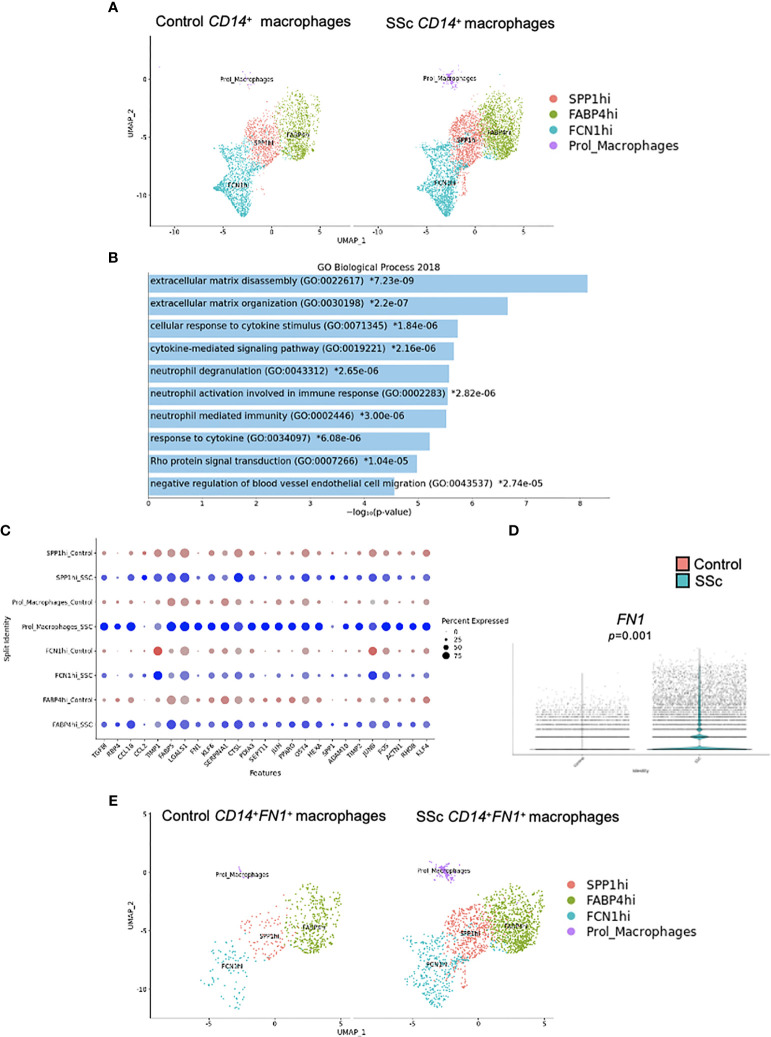
Characteristic of CD14^+^ macrophages in SSc-ILD lung tissues. Four human healthy control (HC) and 4 SSc-ILD lung tissue samples were used for a single-cell (sc) RNA-sequencing analysis according to Valenzi et al. (26). **(A)** UMAP plot visualization of CD14^+^ cells in macrophage clusters in HC and SSc-ILD samples. **(B)** Pathway expression in the specific GO biological process (Enrichr 2018) and the expression of selected fibrotic-related genes **(C)** upregulated in SSc-ILD CD14^+^fibronectin (FN1)^+^ cells compared to HC CD14^+^ cells in lungs. **(D)** *FN1* expression in SSc-ILD CD14^+^ cells compared to HC CD14^+^ cells in lungs (N=4, *Mann Whitney-U-test)*. **(E)** UMAP plot visualization of CD14^+^ cells in macrophage clusters in HC and SSc-ILD samples. UMAP, uniform manifold approximation and projection; SSc-ILD, systemic sclerosis-associated interstitial lung disease.

Transcriptomic analysis of an already published dataset of lung tissue from 4 HC and 4 SSc-ILD patients has been performed (26). CD14^+^ cells were visualized in all macrophage clusters (FCN1hi, SPP1hi, FABP4hi and proliferating macrophages) (**Figure 4A**), determined as previously shown (26). Pathway analysis revealed enrichment of fibrosis-associated processes, such as “Extracellular matrix disassembly”, “Extracellular matrix organization”, “Cellular response to cytokine stimulus” and “Cytokine-mediated signalling pathway” in SSc CD14^+^ cells (**Figure 4B**). At the transcription level, we noticed upregulation expression of several profibrotic genes, including *TGFβ1*, *TIPM1*, *TIPM2, FN1* and *ADAM10* (**Figure 4C**). Importantly, in all macrophage clusters in SSc lungs, we observed upregulated expression of *FN1* in CD14^+^ cells (**Figures 4D, E**).

### Profibrotic Cytokine Stimulation Induces Differentiation of Monocytes Into Myofibroblast-Like Cells

**Figure 5:**
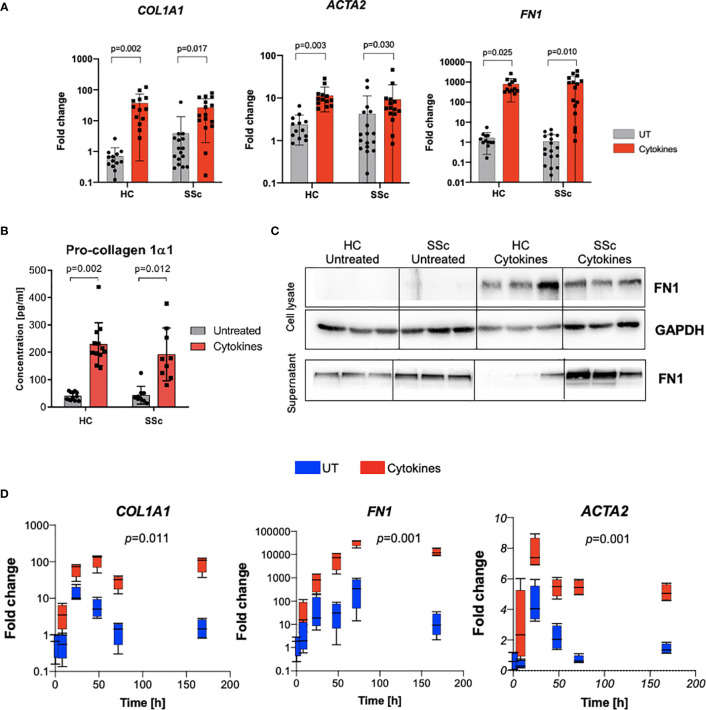
Profibrotic stimulation induces differentiation of monocytes into fibroblast-like cells. **(A)** mRNA expression of *COL1A1, ACTA2 and FN1* after profibrotic cytokines stimulation (TGFβ, IL-4, IL-10, IL-13 [10 ng/ml each]) of HC and SSc monocytes (N=13, *two-way ANOVA with Benjamini, Krieger and Yekutieli post-hoc test*). **(B)** ELISA measurement of Pro-collagen 1α1 concentration in supernatants from CD14^+^ monocytes after profibrotic cytokines stimulation (TGFβ, IL-4, IL-10, IL-13, [10 ng/ml each]) (N=13, *two-way ANOVA with Benjamini, Krieger and Yekutieli post-hoc test*). **(C)** Western blot assessment of fibronectin level in cell lysates and supernatants from CD14^+^ monocytes stimulated with profibrotic cytokines (TGFβ, IL-4, IL-10, IL-13, [10 ng/ml each]) (N=3). **(D)** Time-dependent mRNA expression of *COL1A1, ACTA2 and FN1* after profibrotic cytokines stimulation (TGFβ, IL-4, IL-10, IL-13 [10 ng/ml each]) of HC monocytes at following time points: 0, 8, 24, 47, 72 and 168h (N=4, *two-way ANOVA with Tukey’s multiple comparison test, p values for row factor*).

Bone marrow-derived cells have been proposed as one of the sources of myofibroblasts. Therefore, next, we evaluated the potential of CD14^+^ monocytes to differentiate into myofibroblast-like cells in response to profibrotic stimulation with TGFβ, IL-4, IL-10 and IL-13 cytokines. We observed the induction of profibrotic gene expression including *ACTA2*, *COL1A1* and *FN1* (**Figure 5A**). Additionally, stimulated monocyte-derived cells secreted ECM components: type I collagen and fibronectin (**Figures 5B, C**). Time-dependent profibrotic stimulation with TGFβ, IL-4, IL-10 and IL-13 cytokines revealed that CD14^+^ monocytes upregulated profibrotic gene expression already after 24h and sustain these levels up to 7 days (**Figure 5D**). Of note, we did not observe any difference in the differentiation potential between CD14^+^ monocytes obtained from SSc patients and healthy controls.

### Co-Culture With Dermal Fibroblasts Enhances Profibrotic Phenotype of CD14^+^ Monocytes

**Figure 6:**
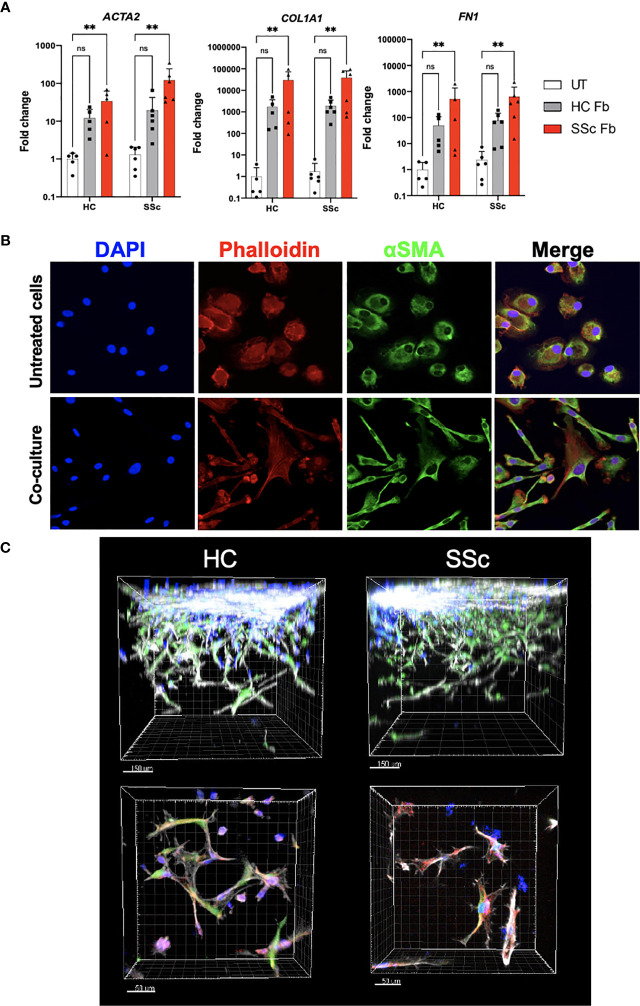
Profibrotic microenvironment induces differentiation of monocytes into fibroblast-like cells. **(A)** mRNA expression of *ACTA2*, *COL1A1 and FN1* in CD14^+^ monocytes from HC and SSc patients after co-culture with HC and SSc skin fibroblasts (HC N=5, SSc N=6, two-way ANOVA with Uncorrected Fischer’s LSD post-hoc test, **p < 0.005). **(B)** Representative images of immunofluorescence staining of CD14^+^ monocytes with and without co-culture with fibroblasts (blue– DAPI nuclear staining, red– Phalloidin staining, green- α-SMA staining). **(C)** Representative images of immunofluorescence staining of HC and SSc CD14^+^ monocytes co-cultured with fibroblasts in 3D hydrogel model (blue– monocytes stained with Cell Trace Violet, green– fibroblasts stained with CFSE, red– α-SMA staining, grey- Phalloidin staining). ns, not significant.

We hypothesized that the microenvironment might play a determinant role in the profibrotic response of CD14^+^ monocytes. To test this hypothesis, we co-cultured CD14^+^ monocytes with side-matched dermal fibroblast from SSc patients and HCs for 7 days. To analyze gene expression, cell types were separated using FACS sorting. We observed that co-culture with dermal fibroblasts induced expression of profibrotic genes *ACTA2*, *COL1A1* and *FN1* in both HC and SSc monocytes. Importantly, we noticed a significant induction of gene expression by SSc fibroblasts (**Figure 6A**).

Further, we aimed to observe phenotypic changes of CD14^+^ monocytes after co-cultures in 2D and 3D models. Monocytes, which were sorted out after the 2D co-culture and re-plated in a chamber slide, were stained with phalloidin and anti-αSMA antibody. Compared to monocytes cultured alone, cells after the co-culture acquired spindle shape, however, they failed to form stress fibers (**Figure 6B**). Similar characteristics were observed for monocytes in 3D co-culture (**Figure 6C**). Taken together, the fibroblast microenvironment induced a change in monocyte gene expression and morphology towards myofibroblast-like cells.

### TGFβ Is a Key Regulator of Profibrotic Gene Expression in Monocyte-Derived Myofibroblast-Like Cells

**Figure 7:**
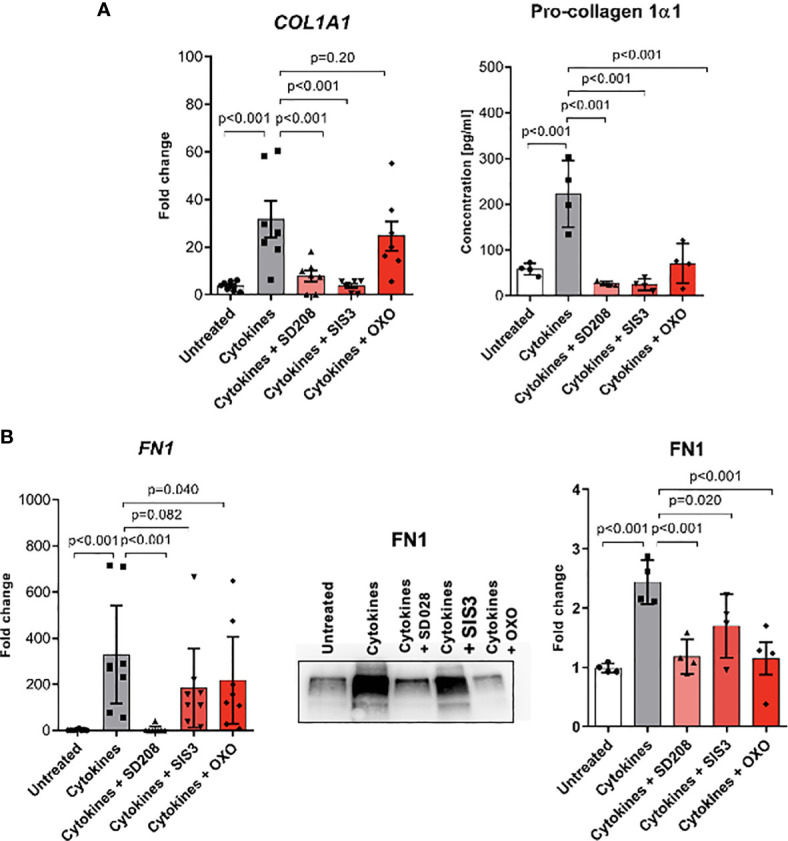
Monocyte-to-fibroblast-like cells differentiation is dependent on TGFβ signalling pathways. **(A)** *COL1A1* mRNA expression level and ELISA measurements of Pro-collagen 1α1 protein level in supernatants after profibrotic cytokine stimulation (TGFβ, IL-4, IL-10, IL-13 [10 ng/ml each]) and treated with TGFβ signalling pathway inhibitors [SD208 and (5Z)-7-Oxozeaenol (OXO) 1 μM, SIS3 2 μM] (N=4-7, *one-way ANOVA with Benjamini, Krieger and Yekutieli post-hoc test*). **(B)** *FN1* mRNA expression level and Western Blot analysis, and corresponding densitometry of Fibronectin protein level in the supernatants after profibrotic cytokine stimulation (TGFβ, IL-4, IL-10, IL-13 [10 ng/ml each]) and treated with TGFβ signalling pathway inhibitors [SD208 and (5Z)-7-Oxozeaenol (OXO) 1 μM, SIS3 2 μM] (N=4-7, *one-way ANOVA with Benjamini, Krieger and Yekutieli post-hoc test*).

Fibrotic processes might be driven by various stimuli; however, TGFβ is regarded as a pivotal mediator during SSc. To analyze the role of TGFβ signalling in monocyte-to-myofibroblast-like cell differentiation, we inhibited selected TGFβ downstream pathways in CD14^+^ monocytes treated with the profibrotic cytokine cocktail (TGFβ, IL-4, IL-10 and IL-13). Treatment with the TGFBR1 kinase inhibitor SD-208 completely abolished expression of the ECM components collagen 1 and fibronectin on both mRNA and protein levels (**Figure 7**). Interestingly, TGFβ-induced expression of *COL1A1* was suppressed by the inhibitor of canonical SMAD-dependent pathway SIS3, but not by inhibition of TAK1-dependent non-canonical pathway with 5z-7-oxozeaenol (OXO). On the other hand, blocking of canonical and non-canonical TGFβ downstream pathways was sufficient to decrease expression and secretion of fibronectin (**Figure 7B**).

## Discussion

Our data demonstrated that human peripheral blood circulating CD14^+^ monocytes exhibited profibrotic phenotype in SSc patients. Similarly, several reports indicated fibroblast-like/circulating Collagen I-producing CD14^+^ monocytes or CD14^-^ fibrocytes as profibrotic cell progeny in SSc (35). Likewise, the alternatively activated macrophages derived from circulating CD14^+^ monocytes have also been considered as profibrotic cells in SSc (36). SSc patients have higher levels of circulating CD34^+^CD14^+^ cells, Collagen-I-producing CD14^+^ monocytes and CD163^+^ monocytes (36). Importantly, monocyte count could be incorporated into the clinical assessment of patients with fibro-proliferative disorders (37), including SSc patients with interstitial lung disease (ILD). Accordingly, the elevated numbers of Collagen-I-producing fibrocytes and profibrotic monocytes in SSc patients were associated with ILD progression and implicated in the pathogenesis of SSc-ILD (36). Circulating Collagen-I-producing fibrocytes have also been coupled with a growing repertoire of human diseases including renal fibrosis (38), cirrhosis (39), nephrogenic systemic fibrosis (40), pulmonary fibrosis (41–43), and asthma (44).

It has been reported that the fibronectin splice variant- fibronectin extra domain A (FnEDA) is an endogenous TLR4 ligand. Importantly, elevated levels of FnEDA were demonstrated in the serum and skin lesions from SSc patients and in mouse model of cutaneous fibrosis (45). In chronic inflammatory diseases and during the healing phase of acute inflammatory reactions, the alternatively activated M(IL-4) macrophages showed significantly upregulated levels of fibronectin that suggest an active role of fibronectin^+^ macrophages in the ECM deposition and tissue remodeling (46). Furthermore, the alveolar macrophages from lungs of SSc-ILD patients displayed elevated levels of fibronectin (47). These reports are in line with our results, which presented the elevated fibronectin levels in circulating CD14^+^ monocytes and CD14^+^ pulmonary macrophages in SSc patients and highlighted the capability of CD14^+^ monocytes to acquire a profibrotic phenotype. Of note, monocyte-to-macrophage differentiation has been linked with the increase production of fibronectin (48). We, therefore, concluded that tissue-infiltrating CD14^+^ monocytes/macrophages can be considered as ECM producers in the pathogenesis of fibrosis in SSc. All these data point to fibronectin as a potential target in therapeutic strategy in SSc.

Monocyte infiltration into the tissue affects the initiation of fibrotic processes in the skin and internal organs in SSc (49–51). Our data clearly presented infiltrating CD14^+^ monocytes in the collagen-rich area in SSc-ILD lungs, myocardium of SSc patients with inflammatory dilated cardiomyopathy (iDCM) and in SSc skin (27). Increased numbers of spindle-shaped CD14^+^CD34^+^collagen-I^+^ cells were found in the lungs of SSc-ILD patients (36). The bone marrow origin of fibroblasts or myofibroblasts in different tissues under homeostasis and disease condition has been debatable for many years now. *In vivo* data, with the use of cutaneous wound mouse model, confirmed that at least a part (15%-20%) of the spindle-shaped dermal fibroblasts were bone marrow origin (52). In contrast, another report suggested that bone marrow-derived progenitors contributed to the inflammatory cell pool infiltrating the wound area; however, they did not differentiate into dermal (myo)fibroblasts at the wound site (53, 54). In the bleomycin-induced skin fibrosis model, a significant number of CD45-positive collagen-producing cells of bone marrow origin contributed to collagen production during dermal fibrogenesis but not under homeostasis, indicating bone marrow originated cells as a cell source for (myo)fibroblasts under disease condition (55). Similarly, in bleomycin-induced lung fibrosis mouse model bone marrow-derived progenitors gave rise to collagen-producing cells but not to αSMA^+^ myofiboblasts in the inflamed/fibrotic lungs (56, 57). Oppositely, in the renal fibrosis the inflammatory macrophages, characterized by alternatively activated macrophage markers, acquired fibroblast phenotype by collagen I and αSMA expression and actively contributed to the fibrogenesis (58).

Nevertheless, the transition of bone marrow-derived monocytes or macrophages (mainly alternatively activated macrophages) into functional myofibroblasts has been mediated by canonical SMAD2/3-dependent or non-canonical TAK-1-dependent TGFβ signal (24, 59–61). Accordingly, our results confirmed that both canonical and non-canonical TGFβ pathways were important to obtain the fibroblast-like phenotype of circulating CD14^+^ monocytes, indicating the broad treatment options.

The pathophysiology of SSc is closely related to the activated TGFβ-dependent pathway. The levels of a latent, not-active form of TGFβ is similar in SSc and healthy serum samples. Active TGFβ serum levels were significantly higher in SSc, mainly in dcSSc, patients and correlated with clinical manifestations (digital ulcers, lung fibrosis, positive antitopoisomerase I and higher modified Rodnan score) (62). These results indicate TGFβ as a potential marker of fibrotic and vascular involvement in SSc on one side, and on the other side, it reflects an altered microenvironment, which may predispose circulating monocytes towards an activated and profibrotic phenotype. Accordingly, we used this cytokine, among others, to activate circulating monocytes towards a profibrotic phenotype. As expected, CD14^+^ monocytes stimulated with TGFβ, IL-4, IL-10 and IL-13 or co-cultured in 2D and 3D model with human dermal fibroblasts were able to adopt a functional myofibroblast-like phenotype that may indicate these cells as one of the cellular sources of profibrotic cells upon their entrance into the tissues. The previous report indeed showed that GM-CSF, IL-4 and endothelin-1 alone or in combination induced myofibroblast-like differentiation of circulating SSc and healthy monocytes (63). Additionally, the profibrotic phenotype of circulating CD14^+^ monocytes from SSc-ILD patients was confirmed by the expression of CD163 and boosted secretion of CCL18 and IL-10 in response to pro-inflammatory stimuli (36). However, as in our stimulation conditions, monocytes from SSc patients showed no differences in acquiring more pronounced profibrotic phenotype compared to monocytes from healthy donors. Therefore, we assume that under inflammatory conditions (i.e. in SSc) more circulating already activated monocytes are recruited to the injury site, where they play a profibrotic role, either by the transition into fibroblast-like cells or by the production of profibrotic stimuli. In line with this hypothesis, Bhandari et al. demonstrated that soluble factors in the local milieu are crucial for pro-fibrotic activation of SSc macrophages (measured as secretion of CCL2, IL-6, TGFβ), arisen from circulating monocytes either stimulated with SSc sera or conditioned media from indirect co-culture with dermal SSc fibroblasts (64). These results point to monocytes and monocyte-derived macrophages as probable key players in fibrogenesis in SSc, suggesting targeted cell therapeutic options as feasible and favorable in SSc.

Besides a known and active role of IL-6 and TGF-β signalling in the pathogenesis of SSc, there is increasing evidence that also Th-2 cytokines: IL-4 and IL-13, are involved in the pathology of SSc (65, 66). Both cytokines relate to profibrotic responses, including connection with increased expression of novel myofibroblast marker: periostin, a matricellular protein important in fibrogenesis (67). Based on our data, the combination of TGFβ, IL-4, IL-10 and IL-13 was sufficient to obtain a fibroblast-like phenotype by circulating CD14^+^ monocytes, indicating that IL-4/IL-13 targeted therapy might be an attractive option against fibrogenesis in SSc (68). Indeed, a randomized, double-blind, placebo-controlled, 24-week, phase II, proof-of-concept study (trial registration number: NCT02921971) with romilkimab (a bispecific immunoglobulin-G4 antibody that binds and neutralizes IL-4/IL-13; SAR156597) in early dcSSc patients revealed a beneficial effect of this treatment on skin disease changes, i.e. statistically significant decrease in mRSS from baseline to week 24 versus placebo (69).

Given the alerted activation status of circulating CD14^+^ monocytes and their ability to accumulate in the affected organs in SSc, we believe that further research should focus on finding even more targeted therapies, which may invert the activation status of monocytes in the circulation.

## Data Availability Statement

The RNAseq datasets for this study can be found in the Gene Expression Omnibus under the accession numbers: GSE157840, GSE128169.

## Ethics Statement

Human blood samples and skin biopsies collection were approved by the local ethics committee of the Canton Zurich (KEK-ZH 515, PB-2016-02014, KEK-Nr 2018-01873). All study subjects provided written informed consent. The experiments with re-use of human material were approved by Swissethics (KEK-Nr 2019-00058, KEK-Nr 2018-01873) and were performed in conformity with the principles outlined in the Declaration of Helsinki. The animal study was reviewed and approved by Commission on Animal Experimentation of the Canton Zurich.

## Author Contributions

GK directed the project and obtained funding. MR, AH, IK, SJ, and GK designed, analyzed, and interpreted experiments. MR, AH, IK, JS, and VM performed the experiments. CF-B and KK provided heart and lungs biopsies. MR, GK, and PB wrote the manuscript. OD, CF-B, ME contributed to final corrections of the drafted manuscript. All authors contributed to the article and approved the submitted version.

## Funding

GK acknowledges support from the Swiss National Science Foundation (310030_152876/1; 310030_175663), Swiss Life Foundation and Swiss Heart Foundation.

## Conflict of Interest

OD had consultancy relationship and/or has received research funding from Actelion, Acceleron Pharma, AnaMar, Bayer, Baecon Discovery, Blade Therapeutics, Boehringer, CSL Behring, ChemomAb, Curzion Pharmaceuticals, Ergonex, Galapagos NV, GSK, Glenmark Pharmaceuticals, Inventiva, Italfarmaco, iQvia, medac, Medscape, Mitsubishi Tanabe Pharma, MSD, Roche, Sanofi, UCBin the area of potential treatments of scleroderma and its complications. In addition, OD has a patent mir-29 for the treatment of systemic sclerosis issued (US8247389, EP2331143).

The remaining authors declare that the research was conducted in the absence of any commercial or financial relationships that could be construed as a potential conflict of interest. 

## Publisher’s Note

All claims expressed in this article are solely those of the authors and do not necessarily represent those of their affiliated organizations, or those of the publisher, the editors and the reviewers. Any product that may be evaluated in this article, or claim that may be made by its manufacturer, is not guaranteed or endorsed by the publisher.
