# Supplementary material for: Elevated Fibronectin Levels in Profibrotic CD14+ Monocytes and CD14+ Macrophages in Systemic Sclerosis
Source: Front Immunol. 2021 Aug 24;12:642891. doi: 10.3389/fimmu.2021.642891 (PMC8421541; doi:10.3389/fimmu.2021.642891)
Supplement: Supplementary file 1 [file DataSheet_1.pdf]

SUPPLEMENTARY MATERIAL

(Manuscript ID: 64289)

Methods

RNA extraction

Total RNA was isolated using the *Quick*-RNA Microprep isolation kit (Zymo research). Directly after monocyte treatment *in vitro*, cells were washed with PBS and lysed in RNA lysis buffer (Zymo research). An equal volume of absolute ethanol (Millipore) was added and mixed. Lysates were further processed on the columns. Genomic DNA was removed by DNase I treatment. RNA was washed twice and eluted in 10-15 µl of nuclease-free water (Promega). RNA concentration and purity were assessed on NanoDrop 1000 (Thermo Fisher Scientific).

RNA sequencing and data analysis

For RNA sequencing, RNA was isolated from SSc patients and healthy controls (as described previously in Supplementary Table S1 in (1)), and RNA Integrity Number (RIN) was assessed by Tape Station (Agilent). Samples with RIN≥8 were further processed. RNA sequencing was performed by the Functional Genomics Centre Zurich. From 100 ng of total RNA, polyA libraries were prepared using the Illumina TruSeq RNA Stranded mRNA library Kit. Sequencing was performed on the Illumina HiSeq 4000 platform. Quality of the sequencing was controlled by FastQC package. Reads were aligned to the genome using the STAR algorithm. Gene expression profiles were next calculated by the FeatureCount algorithm. For differentially expressed genes, the DeSEQ2 algorithm was used with a threshold of minimum 10 reads for a transcript to be considered as present. Pathway enrichment analysis of

differentially expressed genes ($p \leq 0.01$, $\log_2$ratio ≥ 0.5) was performed by the Metacore software.

RT-qPCR

For reverse transcription, 200-300ng of total RNA was used. The reaction was performed using MultiScribe reverse transcriptase (Thermo Fisher Scientific), random hexamers and RNAase inhibitor (both Roche). Subsequently, the qPCR reaction was performed using the SYBR green GoTaq qPCR master mix (Promega) on Agilent Stratagene Mx3005P qPCR instrument. Sequences of primers are listed in Supplementary Table 1. Relative gene expression was calculated using the $2^{-\Delta\Delta Ct}$ method. GAPDH was used as the reference gene.

Supplementary Table 1. Human primes used for RT-qPCR analyses

Gene	***Forward Primer (5' -> 3')***	***Revers Primer (5' -> 3')***
FN1	TGCCACTGTTCTCCTACGTGG	GGAGAATTCAAGTGTGACCCTTG
ACTA2	ACAGAGTATTTG CGCTCCG	CCGACCGAATGCAGAAGGA
COL1α1	CAGCCGCTTCACCTACAGC	TTTTGTATTCAATCACTGTCTTGCC
GAPDH	GGGAAGCTTGTCATCAATGGA	TCTCGCTCCTGGAAGATGGT
RPLPO	ACACTGGTCTCGGACCTGAGAA	AGCTGCACATCACTCAGAATTTCA
TGFβR2	CTCAACCACCAGGGCATCC	GATGCTCCAGCTCACTGAAG
MMP9	CTTTGAGTCCGGTGGACGAT	AATCGCCAGTACTTCCCATC
JUN	CCAACTCATGCTAACGCAGC	TCTCTCCGTCGCAACTTGTC
COL18α1	TGGTCTACGTGTCGGAGCA	GCCTCGTTCGCCCTTAGAG
COL9α3	TTCAGTGCCCAAGTATCTGC	TCGCCTTTGTAGCCAGTG
WNT5β	AGACTGGCATCAAGGAATGC	GTCTCTCGGCTGCCTATCTG

Protein extraction and Western blotting

After the stimulation with cytokines cells were washed once with ice-cold PBS, collected, centrifuged and lysed for 30 minutes on ice in RIPA buffer (Sigma) containing proteases and phosphatases inhibitors (Roche). Equal amount of the protein was loaded and separated by SDS-PAGE electrophoresis, followed by wet transfer on the nitrocellulose membrane (GE Healthcare). The membrane was further incubated for 1 hour in blocking buffer (5% BSA in TBS-T). Further, membranes were probed overnight with primary antibodies (Supplementary Table 2) in blocking buffer at 4°C. Further, membranes were incubated (1 hour, room temperature) with secondary HRP-conjugated antibodies. Signal was developed with ECL substrate (SuperSignal West Pico PLUS, Thermo Scientific) and acquired on the Fusion fx (Vilber) device.

Supplementary Table 2. Antibodies used

Target	Clone	Company	Application
CD14	SP192	Sigma (SAB5500040)	IHC
αSMA	1A4	Sigma (A2547)	WB, IF, IHC
Fibronectin 1	Polyclonal	**Abcam (ab2413)**	WB
Phalloidin (for F actin)		Sigma (P1951)	IF
GAPDH	D4C6R	Cell Signalling (2118S)	WB
CD45	30-F11	BP Pharmingen (55039)	IHC
Arginase 1	Polyclonal	Novus Biologicals (BP-36936)	IHC
CD86	195895	Cell Signalling (E5W6H)	IHC
Mannose-R	GR3357417-1	Abcam (ab64693)	IHC
iNOS	2D-B2	R and D Systems (MAB9502)	IHC

ELISA

For the detection of pro-collagen 1α1 DuoSet ELISA (RnD Systems) was used according to the manufacturer's protocol. For Fibronectin 1 detection we used the antibody describerd in Supplementary Table 2. Briefly, 96-well plates were coated with capture antibodies overnight in room temperature and further blocked with 2% BSA in PBS+Tween 20 (0.05%). Between each step, plates were washed three times with PBS-T. The protein standards and samples were applied and incubated for 2 hours. Next, plates were incubated with biotin-conjugated detection antibodies for 2 hours and streptavidin-HRP for 30 minutes in room temperature. Signal was developed with TMB substrate (Thermo Scientific), and 450 nm absorbance was measured on a BioTEK HT plate reader. Concentrations were calculated according to the respective standard curves.

Treatment with pharmacological inhibitors and cytotoxicity assessment

All pharmacological inhibitors used in the project were purchased from Tocris Biosciences. To determine optimal nontoxic concentrations, we performed toxicity tests. $CD14^{+}$ monocytes were incubated with 2-fold dilutions of inhibitors starting from 5 or 10 μM. Cells were incubated for 24h, stained with PI, and cytotoxicity was evaluated by flow cytometry. The highest non-toxic concentration was used in further experiments.

2D and 3D co-culture with dermal fibroblasts

To distinguish cells in both systems, monocytes were stained with Cell Trace Violet (Thermo Scientific) and fibroblasts were stained with CFSE (Biolegend) according to manufacturer's

protocol. For 2D co-cultures, fibroblasts were plated 24h prior the addition of monocytes. Cells were cultured for 7 days and sorted using FACSAria III cell sorter.

For the 3D co-culture model, 3DProSeed® hydrogel microtiter plate (Ectica Technologies) were used. Firstly, stain fibroblasts were plated and allowed to penetrate hydrogels for 24h. Next, monocytes were added. Plates were incubated for 7 days and anti-αSMA/phalloidin (both Sigma) staining was performed. Co-cultures were visualized using Leica SP8 confocal microscope.

Immunohistochemistry, imaging and quantification

Collected tissue samples were washed in PBS and fixed for 16 hours in 4% paraformaldehyde in PBS. Next, tissues were rinsed in distilled water and transferred to 50% ethanol. Biopsies were then dehydrated (three incubations in 80% ethanol for 1 hour, three incubations in 96% ethanol for 1 hour, two incubations in 100% ethanol for 1 hour). Tissues were cleared twice in xylene for 1 hour and subsequently incubated twice in a 56°C paraffin bath for 3 hours. After paraffin embedding, 4μm thick sections were placed on Superfrost Plus slides (Thermo Scientific) and dried overnight. Sections were deparaffinised in xylene for 10 minutes (3 times) and rehydrated by sequential incubations in ethanol solutions (100%, 100%, 96% and 80%) for 3 minutes each. Sections were eventually washed for 5 minutes in distilled water. Antigen retrieval was performed in citrate buffer (10mM Citrate, 0.05% Tween, pH=6), and incubated at 95°C for 15 minutes. Endogenous peroxidases were blocked by 3% H_2O_2 solution for 15 minutes. Unspecific antibody binding was blocked with 10% goat serum in Background Reducing Antibody Diluent (Dako). Endogenous biotin was blocked by Avidin-Biotin Block kit (Vector Laboratories). Sections were incubated with primary antibodies (Supplementary Table 2) in 4°C overnight. The biotinylated secondary antibodies (Vector Laboratories) were incubated for 30 minutes at room temperature, followed by 30 minutes incubation with

VECTASTAIN Elite ABC kit (Vector Laboratories). Staining was developed using Vector DAB or Vector Red (Vector Laboratories) followed by a counterstaining of nuclei for 1 minute in Mayer's haematoxylin solution (J.T Baker). All stainings were mounted using Pertex mounting medium (Dako). CD14 and pro-collagen 1α1 co-staining was performed by Sophistolab AG (Switzerland). Full slide images were acquired using Zeiss Axio Scan Z1 sclidescanner. ImageJ software was used for relative quantification of the signal in the sections. For CD45 staining analysis, the "HDAB" plug-in was applied, while for Picrosyrius Red and α-SMA "Fast Red, Fast Blue" plug-in was used. Deconvoluted images were used to calculate the area of the nuclear staining and the area of the signal of alkaline phosphatase (AP). The value for each section was calculated as the ratio of AP signal to nuclei signal.

Immunofluorescent staining

Cells were seeded in 8-chamber slides (Lab-Tec) at the density of 2500 cells/well, fixed in ice-cold methanol:acetone 7:3 (both Sigma-Aldrich) for 10 minutes at -20°C, washed with PBS and blocked with 10% FBS in PBS for 20 minutes at room temperature. Subsequently, incubation with the primary anti-αSMA antibody (1:100, Sigma-Aldrich) and Phalloidin (1:100, Sigma-Aldrich) (Supplementary Table 2) was performed for 1 hour at room temperature followed by staining with the secondary antibody (goat anti-mouse, 1:400, Thermo Fischer Scientific) and 50 µg/ml of fluorescent labelled phalloidin (Sigma-Aldrich) to visualise stress fibres for 45 minutes at room temperature. Nuclei were counterstained with DAPI solution (1 µg/ml, Roche). Images were acquired with an Olympus BX53 microscope equipped with a DP80 camera.

Reference:

1. Rudnik M, Rolski F, Jordan S, Mertelj T, Stellato M, Distler O, et al. CD52 regulates monocyte adhesion and interferon type I signalling in systemic sclerosis patients. Arthritis Rheumatol. 2021.
